# CRISPR Technology: A Jewish Legal Perspective

**DOI:** 10.5041/RMMJ.10487

**Published:** 2022-10-27

**Authors:** John D. Loike, Rabbi Tzvi Flaum

**Affiliations:** 1 Interim Director of Bioethics, School of Health Sciences and Practice, New York Medical College—Associated with Touro University, Valhalla, NY, USA; 2 Professor of Biology, Touro University, New York, NY, USA; 3 Associate Professor, Judaic Studies, Mashgiach Ruchani, Lander College for Women (Touro College), New York, NY, USA

**Keywords:** CRISPR, gene editing, Jewish legal issues

## Abstract

Clustered regularly interspaced short palindromic repeats (CRISPR) gene editing is an innovative and potentially game-changing biotechnology that can potentially reverse DNA mutations in a tissue-specific manner. In addition, CRISPR is being targeted for xenotransplantation, for increasing human longevity, in animal breeding, and in plant science. However, there are many ethical challenges that emerge from CRISPR technology. This article discusses several positions that relate to these ethical challenges from a Jewish legal perspective. In addition, we present several other applications of CRISPR technology that lack a defined Jewish legal precedent and require rabbinical scholars to address and resolve them in the future.

## INTRODUCTION

One of the most exciting and versatile biotechnologies that scientists are mastering is gene editing using clustered regularly interspaced short palindromic repeats (CRISPR) technology,^1^ which is more than merely a gene editing technology as originally proposed. Genome editing using CRISPR-Cas9 takes advantage of a bacterial CRISPR-Cas system to modify a genome in a targeted manner. Guided by an RNA sequence, the Cas9 enzyme breaks DNA at a specific site. Normally, cellular repair of these breaks is imprecise; however, with CRISPR, humankind now has the power to rewrite the sequences of at least small regions of the genome since the repair pathways can be engineered to correct mutations or insert new or corrected genes.

This technology now has many potential applications in diagnosing diseases, medical treatments, animal breeding, and agriculture. In this article, we first present several exciting innovative applications of CRISPR that have emerged within the past two years. We then discuss some of the ethical challenges in using CRISPR technology from a Jewish bioethical perspective. For some of these challenges, we have identified rabbinical opinions and precedents, but other ethical issues remain complex and need to be further analyzed by our rabbinical scholars.

The CRISPR technology emerged from successful scientific efforts to sequence prokaryotic (e.g. bacterial) DNA, thereby opening the door to genetically altering or editing almost any part of the genome of humans and other organisms.^2^ Emmanuelle Charpentier and Jennifer Doudna, who made key discoveries in the field of DNA manipulation with the CRISPR-Cas9 system, were awarded the Nobel Prize in Chemistry for their work on CRISPR in 2020.

**Figure 1:**
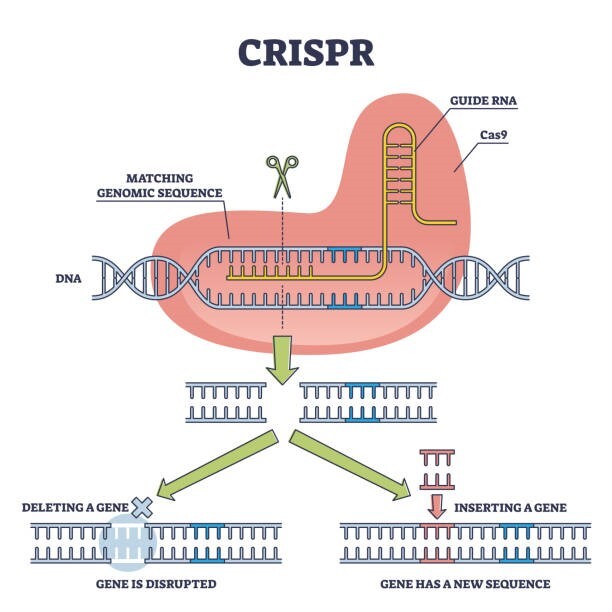
The CRISPR-Cas9 System.

There are two essential biological components of CRISPR technology (Figure 1).^2^ The first set of components are proteins called CRISPR-associated proteins (Cas) that function as molecular scissors to initiate double-stranded breaks in DNA. The second set of components are guide RNAs, made up of two parts: CRISPR RNA (crRNA) and trans-activating CRISPR RNA (tracrRNA). The guide RNAs target the exact site on the DNA where Cas will make its breaks. There are three main types of CRISPR-mediated editing mechanisms to generate genetic manipulations. First, CRISPR can cause a single cut in the DNA, disrupting the original DNA sequence and causing gene inactivation. Second, a larger fragment of DNA can be deleted by using two guide RNAs. After cleavage at each DNA site, cellular repair processes join the separate ends of the remaining DNA fragment. Third, a DNA template can be added to the CRISPR machinery to allow the cell to correct a gene or even insert a new gene. An important research objective in programming CRISPR was understanding the cell’s natural repair mechanisms to edit the genome.^3^

## POTENTIAL CLINICAL APPLICATIONS

The CRISPR technology was adapted from observing bacteria that use this process to cut and destroy invading viruses.^2^ Bacteria, like any living cell, can be infected with viruses and have the capacity to protect themselves from recurring viral infections. Bacteria that have survived one round of a viral infection can capture snippets of DNA from invading viruses and use them to create DNA segments known as CRISPR arrays. The CRISPR arrays allow the bacteria to “remember” newly invading viruses. When a bacterium is re-infected, it recognizes the newly invading viral DNA and produces RNA segments from the CRISPR arrays to target the DNA of the invading virus. A normal protein encoded by bacteria, called Cas9, is then turned on to cut the viral DNA and destroy the capacity of the virus to replicate. In a broad sense, CRISPR represents a bacterial immune system.

As stated above, in using CRISPR-Cas9, the DNA sequence of any gene in the human genome can be modified in ways that can knock out its function completely, introduce or correct a very specific mutation, or add an extra sequence. For example, CRISPR technology can be applied in humans to destroy invading viruses.^4^ The Broad Institute of the Massachusetts Institute of Technology and Harvard University^5^ have programmed a CRISPR system to destroy three different single-stranded RNA viruses in human embryonic kidney cells, with the end target to create new antiviral therapies to treat diseases such as Ebola, Zika, HIV, or COVID-19.

Another future application of CRISPR involves creating appropriate template DNAs that allow the cell to specifically repair the Cas-generated breaks. The goal is to program Cas9 using a guide RNA to find a specific mutation that causes a genetic disease and cut the genome at this location. The mutation can then be replaced, using the template DNA, with the DNA sequence that encodes a healthy individual. In this manner CRISPR can be used to correct genetic diseases^6^ such as cystic fibrosis, Tay–Sachs disease, or Duchenne muscular dystrophy.

The CRISPR technology is not limited to genetic diseases caused by simple mutations. Several of the approximately 7,000–10,000 genetic diseases, such as Huntington’s disease, result from an over-replication of many base pairs (nucleotide repeats) or gene regions in cells. This over-replication results in the generation of abnormal proteins that destroy these cells and cause these diseases. To treat these conditions, CRISPR technology can be employed to cut away the unnecessary multiple genomic repeats to stop the cells from producing these abnormal proteins.

In early 2022, scientists used CRISPR technology to treat a patient with a genetic form of sickle cell anemia.^7^ The patient had a mutation in the gene encoding for the adult form of hemoglobin causing the sickle cell disease. However, in the patient’s embryonic stage, sickle cell disease had not manifested due to the presence of a gene that was turned on and encoded for a fetal form of normally functioning hemoglobin. However, once the patient was born, the embryonic gene was turned off and the adult form of hemoglobin was turned on. Since the sickle cell mutation is located in the adult form of hemoglobin, the patient only experienced all the symptoms of sickle cell disease after birth. Using CRISPR technology, scientists were able to inactivate the “turn-off switch” for fetal hemoglobin, allowing the patient to continue to produce fetal hemoglobin even after birth, thereby mitigating the damaging effects of the mutant form of adult hemoglobin. In this milestone clinical trial, CRISPR created an effective treatment for sickle cell anemia that paves the way to apply CRISPR technologies to treat other diseases.

## INNOVATIVE CLINICAL APPLICATIONS OF CRISPR

Phage therapy is an example of an innovative CRISPR technology that genetically alters bacteriophages (the viruses that invade bacteria) to treat antibiotic-resistant bacterial infections.^8^ The trick here is to introduce a CRISPR system in bacteriophages (the viruses that invade bacteria) so that when they infect the targeted antibiotic-resistant bacteria they will cut and dice the bacterial genome, terminate bacterial DNA replication, and stop the bacterial infection. Bacteriophages are highly specific pathogens for bacteria and do not normally infect human cells, making this procedure relatively safe to treat humans. In fact, over 100 patients in Belgium have been treated with this therapy, with about 70 of these patients showing clear clinical improvement. For example, in March of 2016, a 30-year-old woman was severely injured in a suicide bombing at Brussels airport and developed an antibiotic-resistant bacterial infection triggered by *Klebsiella pneumoniae.* Because this bacterium was resistant to almost all drugs, the physicians could not control her infection. In February 2018, she was treated with a CRISPR genetically engineered bacteriophage that specifically invades this bacterium, and in combination with antibiotics her condition improved within weeks such that she is now able to walk again. In the three years after her treatment, the bacterial infection has not returned.^9^

### Cancer

Many cancers are caused by single base-pair changes in specific genes, such as BRCA1 or BRCA2, and these mutations represent potential repair targets for CRISPR. The main problem in using CRISPR to treat cancer is that only the specific cancer cells must be targeted, without including any healthy cells. However, CRISPR in combination with established immunotherapies, such as CAR-T, appears to be a more effective protocol, especially in tumor eradication.^1^ In addition, CRISPR can be designed to delete key genes in the patient’s immune system to better repopulate the bone marrow with engineered lymphocytes that specifically kill the tumor cells, or to avoid tissue rejection when applying CAR-T to treat leukemias. Finally, CRISPR can be used to convert the mutant cancer protein that causes breast cancer to the normal protein.^10^

### Treating Other Diseases

Using a novel CRISPR system, scientists have targeted CRISPR to the central nervous system in mouse models of amyotrophic lateral sclerosis and Huntington’s disease to deactivate mutated proteins that cause these diseases.^11^ In these protocols CRISPR detects the mutant mRNA found in the cells affected by these diseases and activates a special Cas protein that destroys the mRNA that encodes for the abnormal protein, thereby preventing the cells from producing the mutant protein.

### Diagnostics

In January of 2022, United PPE America^12^ used a SHERLOCK technique to detect COVID-19 in samples obtained from the nose, mouth, or blood. This inexpensive diagnostic test provided an easy readout within one hour. The SHERLOCK technique uses Cas13 molecular scissors that when activated will cut a reporter RNA molecule added to the sample. If the sample contains a specific viral sequence, then CRISPR will activate Cas13, which then cuts the reporter DNA causing the reporter to appear as a positive line on the test strip. Samples not containing the viral genetic markers will not activate Cas13; the reporter DNA remains intact and will not generate a readable signal.^13^ Not only can SHERLOCK be used to detect COVID-19 or any of its variants, but it can be programmed to detect almost any virus, including the influenza virus.^14^

### Gene Drive Technology

Gene drive technology is a type of genetic engineering protocol that modifies genes that do not follow typical rules of heredity.^15^ For example, scientists can create a breed of mosquitoes that have a unique CRISPR system transplanted into their genome. When these mosquitoes mate with wild-type mosquitoes, the CRISPR system will selectively change a normal gene of the wild-type mosquito to an altered mutant form of the gene that dramatically reduces egg production of the females.^16^ One ultimate goal of gene drive is to eliminate malaria-carrying mosquitos, since the females are the hosts for malaria parasites.

### Xenotransplantation

In January of 2022, physicians in Baltimore performed the first pig to human heart transplant.^17^ The 57-year-old transplant recipient lived almost three months after the procedure. The transplanted heart came from a pig raised by the xenotransplantation company Revivicor (Blacksburg, VA, USA), which used CRISPR technology to alter at least 10 of the pigs’ genes so that their tissues and organs would be *less likely* to be rejected by the human immune system after transplantation. One key CRISPR edit was knocking out several pig-specific antigens to mitigate the human immune response against foreign antigens and adding six human genes that foster immune acceptance of pig organs.^18^ In addition, CRISPR was used in these genetically engineered pigs to inactivate specific pig genes that could cause cancers in humans. This heart transplant followed the first successful pig kidney xenotransplant to a brain-dead woman in October of 2021.^19^ With consent of her family, the brain-dead woman’s body was maintained on a ventilator for 54 hours after receiving the organ, during which time key indicators of a properly functioning kidney were observed. The physicians used a brain-dead person as a precaution to better understand potential side effects of such transplantation. However, this raises a number of ethical issues that merit consideration, some of which are discussed in the appropriate section below.

Xenotransplantation is a rapidly progressing field.^20^ In the future, scientists may be able to use stem cell technology and CRISPR to transplant an organ donor’s stem cells into a genetically engineered pig embryo, resulting in a pig born with the donor’s kidney or heart. The CRISPR technology would be used to turn off the pig’s genes that regulate its kidney development, enabling the pig embryo to use the donor’s reprogrammed stem cells to develop a human kidney with the patient’s cellular “fingerprint”, meaning that very little or even no anti-rejection drug treatment would be required. Unfortunately, only 41,000 organ transplants were performed in the United States in 2021, while over 100,000 patients needed a transplant.^21^ Thus, xenotransplantation using CRISPR technology may become a life-saving procedure for patients lacking a compatible human organ donor.

Some scientists fear that xenotransplantation may increase the risk of triggering a new zoonotic pandemic, where viruses could jump from animals to humans as we have just witnessed with COVID. Again, CRISPR technology may be employed to remove any endogenous virus harbored in the organs of these genetically engineered pigs.^22^

## LONGEVITY

Telomeres are regions of repeated nucleotides capping the ends of chromosomes and playing a crucial role in cellular senescence or biological aging. Telomeres protect the ends of chromosomes from damage. At birth, most humans have a telomere length of about 15,000 nucleotide DNA base-pairs. As humans age and cells divide, these chromosome ends, or telomeres, shorten until they reach the 5,000 base-pair mark. When the telomeres shorten below a critical length, multiple cellular signaling pathways can be initiated that stop the cells from dividing. This cellular senescence can lead to organ failure in the tissues containing these short telomeres. Different species have different telomere lengths, and, in general, increasing telomere length is associated with an increased lifespan of an organism.^23^ Altering the length of telomeres or shortening the decay of the telomeres may provide a unique and important avenue of study in improving both the life expectancy and quality of human life.

Today, CRISPR technologies have developed to the point that scientists can either slow the shortening of telomeres or even lengthen existing telomeres.^24^ In either case, such changes may have profound effects in lengthening human lifespans. In addition to altering telomere length, CRISPR technologies can be used to increase the expression of specific genes, such as SIRT6, that are vital to slow human aging. In 2021, Israeli researchers increased the life expectancy of mice by almost 30% by introducing high levels of the SIRT6 gene, which improved the capacity of these mice to generate energy in the absence of external energy sources.^25^,^26^

## RAISING ETHICAL CONCERNS ABOUT CRISPR

A Chinese biophysicist, He Jiankui used CRISPR-Cas9 to modify the genomes of human IVF-generated embryos with the intention of making them HIV-resistant. He implanted the edited embryos into women resulting in the birth of twins in October 2018 and a third baby in 2019.^27^ Doctor He’s experiments raised international condemnation over his risky use of gene-editing in human embryos before scientists had a clearer understanding of the health risks in using CRISPR. For example, CRISPR can give rise to cell toxicity and genomic instability.^28^ In response to He’s experiments, the Chinese government set up the National Science and Technology Ethics Committee, a high-level group of experts to oversee governance of research ethics. Because Doctor He did not follow established ethical guidelines, a Chinese court sentenced him to three years in prison.

## JEWISH ETHICAL ISSUES

There are three basic Jewish ethical issues in CRISPR technology. The first is identifying the medical and non-medical applications in CRISPR-based treatments.^29^ The second is the issue of “playing God.”^30^ The third relates to the question of whether or not modifying a species with CRISPR should be included in the prohibition of *kilayim* (creating new breeds of animals).^31^–^33^ The first two issues are the focus of this paper.

In general, the rabbinical consensus is that the application CRISPR for medical reasons, such as diagnosis and disease treatment, will take precedence over any possible Jewish ethical objections inherent in the technology. Many rabbinical decisors (arbitrators of the Jewish law) follow the opinion of the famous commentary of the Mishnah, Tiferet Yisrael, which states:

anything where there is no reason to forbid [according to Torah law] is permissible in Jewish law and needs no justification. For the Torah has not enumerated all permissible things, rather the Torah focuses on forbidden laws.^34^

Another important Jewish guideline is the role of human beings to partner with God to improve the world and conquer disease. This guideline is articulated by several biblical commentators such as the Ramban and Rabbi Shimshon Hirsch on verse 7 in Genesis 9:

Be fruitful, and multiply, and replenish the earth, and subdue it; and have dominion over the fish of the sea, and over the fowl of the air, and over every living thing that creepeth upon the earth. (Genesis 9:7)

Ramban^35^ and Rabbi Hirsch^36^ understand this verse to mean that God gave mankind the right to use all natural resources, inanimate and animate, for use to better the world and fight disease. Rabbi Joseph Ber Soloveitchik^37^ expands this position by stating that all stories in the Torah point to a moral lesson to mankind. The description in Genesis that God created the world serves as a moral directive that God instructs man to be creative and use his creative talents to partner with God and preserve and improve the world.

The above-mentioned ethical guidelines serve in conjunction with the critical commandment that everyone (especially physicians) must do whatever is possible to save human lives, and ethically justifies the use of CRISPR in medical therapies and in diagnostic tests. While some secular and religious scholars discuss the possible ethical issue of “playing God,” this is not a Jewish ethical issue in cases where saving lives is critical.

The Jewish legal precedent permitting genetic manipulation for medical purposes arose recently with respect to mitochondrial replacement therapy. About 7 in 100,000 women are born with serious mitochondrial DNA (mtDNA) mutations^38^ that can lead to multiorgan dysfunction.^38^ Unfortunately most of these mtDNA mutations will also affect their children. Mitochondrial replacement therapy (MRT) is a method that can help these women to have healthy children. There are multiple ways to perform MRT. One common method involves transferring the affected mother’s nuclear genetic material from her egg (oocyte) containing the diseased mitochondria into a donor egg with healthy mitochondria and from which the original nuclear genetic material has been removed. The healthy mitochondria in the donor egg enable normal embryological development and prevent transmission of the mitochondrial disease to the child. The genetically reconstituted oocyte can then be fertilized by the husband’s sperm to produce a healthy child.^39^,^40^ Even though generating healthy children from women with mitochondrial diseases involves genetic manipulation and may violate “playing God,” this procedure has not been prohibited by any major Jewish legal decisor.

Another ethical issue that Jewish legal decisors need to address is using CRISPR to enhance the human condition (i.e. non-medical applications), or to create designer babies, i.e. babies with a specific hair or eye color, with high intelligence, or with enhanced athletic skills. Here, the Jewish legal discussions regarding plastic surgery to improve one’s appearance may serve as a Jewish legal precedent to address the use of CRISPR for non-medical applications. Rabbi Eliezer Waldenberg^41^ argues forcefully against pure cosmetic surgery for two reasons. First, the Torah does not give physicians permission to practice their craft in a non-healing context. Second, these procedures violate the commandment that one is not permitted to wound oneself or others for no medical benefit. In contrast, Rabbi Moshe Feinstein interprets the Maimonides’ prohibition of wounding oneself and others as only applying when done in a contentious manner.^42^ Cosmetic surgery is done usually to undo the humiliation being suffered with bad appearances and therefore would not be considered a violation of wounding. In addition, plastic surgery does not usually result in significant health risks and therefore can be permitted with rabbinic approval. Many rabbis also take into consideration psychological reasons in their analysis of Jewish law^43^ and believe that normative appearances such as pierced ears or liposuction are allowed to be done when the patient has legitimate psychological concerns about their appearances. Moreover, cosmetic surgery or dyeing one’s grey hairs are viewed as permissible, according to Jewish law, if the person believes this will help preserve his or her jobs or make them more effective at their jobs. The rabbinical permissibility of allowing cosmetic surgeries creates a rabbinical precedent and offers guidelines to allow use of CRISPR technology for non-medical or enhancement procedures when these enhancements will mitigate psychological or employment issues.

There are several other ethical issues that require rabbinical input. For example, can parents use CRISPR to alter the genes of embryos to have blue eyes or enhance their intelligence? If the parents are currently living in a Scandinavian country where blue eyes and blond hair are the norm, can they use CRISPR to produce children with blue eyes and blond hair so that their children will be more accepted into that society? To date, rabbinical scholars prohibit the use of genetic engineering in creating designer babies where there is no medical benefit (Rabbi Flaum, personal communication).

Another Jewish ethical issue is whether rabbis would allow the use of CRISPR technology to increase the lifespan of human beings. The assumption here is that CRISPR will increase human lifespan without presenting adverse and unexpected health risks. Does the Torah’s perspective that most human beings do not live past the age of 120 represent a Jewish legal directive? Rabbis might argue that prolonging human life provides an opportunity to better serve God and mankind or to make the world a better place. The Biblical obligation to live by the commandments and not to die by them, is strongly emphasized by many Jewish sources.^44^–^50^ Why, then, did God decide to shorten man’s lifespan, making 120 years the upper limit?^51^ In addition, the underlying philosophical principle is that only God decides on the lifespan of human beings.^52^–^55^ Ecclesiastes (3:2a) states: “There is a time to be born and a time to die.” Whether a person can use CRISPR to increase his or her lifespan according to Jewish law has not been fully addressed and will require more input from rabbinical decisors.

The Jewish ethical issue of using CRISPR to develop xenotransplantation is more complex. Rabbinical decisors would need to consider the Jewish mandate of preventing animals from suffering and assess whether xenotransplantation violates the prohibition against animal suffering. Again, the use of xenotransplantation to save human lives will take precedence over the issue of animal suffering and many other potential Jewish ethical issues. It is also important to note that pigs are not the only source for human organs. In 2021, scientists created the first embryos that were part human and part monkey^56^ and kept these embryos alive for 20 days. In rabbinical law, there does not appear to be any difference between using a kosher animal, a pig, or a non-human primate as an organ donor. Another issue is whether or not one should remove one kidney from a pig donor and preserve that animal.^57^,^58^ Again, there is a Jewish legal precedent: pigs can be used as sources for aortic valve replacement,^59^ and there does not appear to be a rabbinical objection to killing these animals for their heart valves. In summary, Judaism might look with favorable eyes upon this procedure to prolong or save the life of a human being who is ill or dying from organ failure.^60^

Nonetheless, there are still other unique Jewish legal challenges in xenotransplantation that our current rabbinical decisors need to consider. First, is it permissible to engage in xenotransplantation technologies to create pigs or mice that produce human sperm or eggs? Sometimes individuals become sterile because of genetics, illness, or radiation/chemotherapy and cannot produce sperm or eggs. With stem cell and CRISPR technologies, scientists have generated mice that produce viable human sperm^61^ or eggs.^62^ To date, these gametes have not been used to generate a human being even though technically they could. One could theoretically extract sperm or eggs from these genetically engineered mice or pigs for *in vitro* fertilization or artificial reproductive technologies to enable sterile people to have healthy children. There may be a precedent in Jewish law stated by the famous Talmudic fourteenth-century commentary by Rabbi Menachem ben Solomon (Hameiri). In his discussion of the Jewish prohibition of using magic, he makes the bold statement:

whatever is achieved through natural means does not constitute “kishuf” [sorcery]. Anything which is done via a natural [biological] act is not included in sorcery. Even if they know how to create beautiful creatures without sexual reproduction—as is known in the books of nature that this is not impossible—it is permitted to do, because anything which is natural is not included in sorcery. And similar to this, [anything] which is medicinal is not [a problem].^63^

In the future there may be situations where it will be permissible, according to Jewish law, to create human beings without normal marital relations, such as parthenogenesis or the use of animal-derived human sperm or human eggs. Thus, all of these innovative reproductive technologies may be permitted according to the Hameiri, because CRISPR and stem cells are part of the normal biological order.

Inherent in any discussion of reproductive technologies, the rabbis must also consider parental status. For example, what is the fatherhood status of a child born from a woman who has been inseminated with human sperm obtained from a chimeric animal that produces human sperm? If the stem cells used to create this chimeric animal came from a Kohen or Levi, does the child inherit the Kohen or Levi status of the stem cell donor? Similarly, what is the Jewish legal opinion on the motherhood of a child born from a woman impregnated using a human oocyte obtained from these chimeric animals? Is that child Jewish? These legal issues need to be expanded to discuss the various Jewish laws to determine which relatives such a child cannot marry. Many rabbinical leaders believe that the Jewish legal status of a child depends both on the status of the woman carrying the embryo and the genetic background of the embryo. Therefore, it is important to consider how these criteria will be applied to human gametes generated from chimeric animals.

Xenotransplantation technology can also be used to generate human neurons in the brains of animals, creating human–animal neural chimeras. Scientists are currently developing these chimeras, as animal models, to study neural development and degenerative neurological disease.^64^ However, these chimeras may blur the distinctions between human beings and non-human animals. At what point will the use of CRISPR to enhance cognition in these chimeric animals transform these “animals” to attain humanoid status? Rabbi Moses Dovid Tendler, Zt’l, cautioned against the research use of such human–animal neural chimeras because it may violate an important Jewish principle of respecting human dignity (*kavod habriut*).^65^,^66^

A case that goes beyond the scope of this article can be derived from the research done on a brain-dead individual, discussed above.^19^ Should Jewish scientists be allowed to transplant a humanized pig organ into a brain-dead individual to perfect a technology that could potentially save hundreds of thousands of lives in the future? Inherent in this case, rabbis must consider whether using brain-dead people constitutes the prohibition of *nivul hames* (defiling the deceased), and how to apply the definition of death according to Jewish law, i.e. cessation of cardiac function or brain stem inactivity.^67^,^68^

## CONCLUSIONS

Clearly, there is a great deal of potential in developing CRISPR technology in therapeutic medicine, animal science, and plant science. In this article we have just begun to analyze how Jewish law will resolve the many ethical challenges of CRISPR technologies. We have not discussed the Jewish legal issues related to the use of these technologies in altering agricultural organisms or creating organisms with new genotypes or phenotypes that these species do not normally carry. For example, CRISPR can be used to insert the pig’s gene for muscle myoglobin into a cow’s embryo so that the meat from a kosher cow will taste more like pork. We leave those issues for a future publication.

The initiation of clinical studies utilizing CRISPR technology to correct genetic mutations also opens a new door in genetic counseling. Couples who engage in prenatal genetic counseling must deal with serious Jewish legal issues. For example, not all rabbinical decisors will allow a couple to terminate the pregnancy if the embryo is found to express genetic mutations. Moreover, many rabbis discourage couples from engaging in prenatal diagnosis. As CRISPR technology will hopefully develop into a safer and more effective treatment of genetic diseases, prenatal diagnosis may not require the couple to deal with the ethical issues of pregnancy termination, but may allow couples to apply CRISPR technology to treat their genetically affected baby.

Herein we have also discussed situations where there are rabbinical precedents. In addition, we highlighted other situations that will require Jewish legal scholars to address the ethical challenges. In several of these cases, it may turn out that there are no specific Torah or rabbinical prohibitions that relate to CRISPR technology—just deciding under what conditions we can use CRISPR. Finally, all rabbinical scholars recognize another important principle in Jewish law—that any Jewish legal decision must follow both the letter of the law and the spirit of the law.^69^

## Abbreviations

Cas: CRISPR-associated proteins
CRISPR: clustered regularly interspaced short palindromic repeats
crRNA: CRISPR RNA
tracrRNA: trans-activating CRISPR RNA
